# Cercarial Dermatitis Transmitted by Exotic Marine Snail

**DOI:** 10.3201/eid1609.091664

**Published:** 2010-09

**Authors:** Sara V. Brant, Andrew N. Cohen, David James, Lucia Hui, Albert Hom, Eric S. Loker

**Affiliations:** Author affiliations: University of New Mexico, Albuquerque, New Mexico, USA (S.V. Brant, E.S. Loker);; Center for Research on Aquatic Bioinvasions, Richmond, California, USA (A.N. Cohen);; Alameda County Vector Control Services District, Alameda, California, USA (D. James, L. Hui, A. Hom)

**Keywords:** Schistosome, cercarial dermatitis, swimmer’s itch, marine snail, nonnative, Haminoea, San Francisco Bay, California, research

## Abstract

Cercarial dermatitis (swimmer’s itch) is caused by the penetration of human skin by cercariae of schistosome parasites that develop in and are released from snail hosts. Cercarial dermatitis is frequently acquired in freshwater habitats, and less commonly in marine or estuarine waters. To investigate reports of a dermatitis outbreak in San Francisco Bay, California, we surveyed local snails for schistosome infections during 2005–2008. We found schistosomes only in *Haminoea japonica*, an Asian snail first reported in San Francisco Bay in 1999. Genetic markers place this schistosome within a large clade of avian schistosomes, but do not match any species for which there are genetic data. It is the second known schistosome species to cause dermatitis in western North American coastal waters; these species are transmitted by exotic snails. Introduction of exotic hosts can support unexpected emergence of an unknown parasite with serious medical or veterinary implications.

## Abstract 2

TOC summary: Introduction of exotic hosts can support unexpected emergence of unknown parasites.

One consequence of introduction of exotic species is possible establishment of new host–parasite associations, potentially resulting in emergence of new diseases (*1**–**3*). Exotic parasites can be introduced into new locations along with their exotic host species (*1**,**4*), sometimes causing extinction of indigenous parasites (*5*). Newly introduced parasites can extend their host ranges into related indigenous host species (*6**,**7*), or exotic hosts may play new roles in the transmission of indigenous parasites (*8*). Parasites newly supported by these exotic hosts can assume considerable human or animal roles as emerging disease agents (*9*).

San Francisco Bay has been the site of numerous well-documented introductions of exotic species (*10**–**12*). We document an outbreak of human cercarial dermatitis in San Francisco Bay that was related to the recent introduction of an exotic snail, the Japanese bubble snail *Haminoea japonica* Pilsbury 1895 (Cephalaspidea: Haminoeidae), which serves as the intermediate host of a schistosome that is responsible for the now annual dermatitis outbreaks.

Cercarial dermatitis (swimmer’s itch) is caused by penetration of human skin with cercariae of schistosome parasites; the condition is common and recurrent in freshwater habitats worldwide. Adult schistosomes typically live in mesenteric blood vessels of birds or mammals and produce eggs that pass from the host in feces. The eggs then hatch and release miracidia, which penetrate and develop in an appropriate species of an intermediate snail host. Snail infections culminate in asexual production of numerous cercariae, which are regularly released into the water where they seek to penetrate the skin of a definitive vertebrate host. Penner reported an association between human dermatitis and a marine schistosome (*13*). He established that *Littorina keenae* Rosewater 1978 (Hypsogastropoda: Littorinidae) syn. *L. planaxis* snails collected along the rocky shores of southern California released schistosome cercariae that caused dermatitis in experimentally exposed human volunteers. Documented cases (*1**,**14**–**21*) have been attributed to species of *Austrobilharzia* Johnston 1917, the adults of which most commonly infect gulls and shorebirds (*14*).

A cercarial dermatitis outbreak in San Francisco Bay was reported in 1954 (*1*). The Bureau of Vector Control of the California Department of Health Services identified the cercariae as *Austrobilharzia variglandis* (Miller and Northrup 1926) collected from the eastern Atlantic mudsnail *Ilyanassa obsoleta* (Say 1822) (Hypsogastropoda: Nassariidae) syn. *Nassarius obsoletu*s at Robert Crown Memorial Beach in 1955 and 1956 (*1*). *I*. *obsoleta* snails were accidentally introduced into San Francisco Bay in commercial shipments of Atlantic oysters and were observed in the bay in 1907 (*10**,**11*). *A. variglandis* schistosomes had been identified as the cause of cercarial dermatitis in coastal waters in the northeastern United States (*14*).

After the 1954 outbreak, no additional cases of cercarial dermatitis were reported in San Francisco Bay or elsewhere on the Pacific Coast until the summer of 2001 when 36 cases of cercarial dermatitis were reported at Crescent Beach in Surrey, British Columbia, Canada; 44 cases were reported in 2002 (*20*). The agent again appeared to *A. variglandis* schistosomes carried by *I. obsoleta* snails, which are extremely abundant at Crescent Beach. The snail was accidentally introduced into British Columbia in the oyster trade and was first observed in the region in 1952 (*20*).

In June 2005, cercarial dermatitis appeared again in San Francisco Bay. The Alameda County Department of Environmental Health received ≈90 reports of skin irritation that occurred after water contact at Robert Crown Memorial Beach where the 1954 outbreak had occurred (The Swimmer Itch Hotline, www.acgov.org/aceh/recreational/beaches.htm). Although it was initially assumed that *A. variglandis* schistosomes, carried by *I. obsoleta* snails, were the causative agent, preliminary investigations suggested that the intermediate host involved was another exotic species, *Haminoea japonica*, from Asia. This snail was observed in San Francisco Bay in 1999 (*22*) and on the eastern shore of the bay, near Robert Crown Memorial Beach, in the summer of 2003, where it has become extremely abundant (A.N. Cohen, unpub. data). We describe the morphologic and genetic features of cercariae obtained from *H. japonica* snails and discuss possible transmission pathways of this parasite in San Francisco Bay.

## Methods

### Specimen Collection

**Figure 1:**
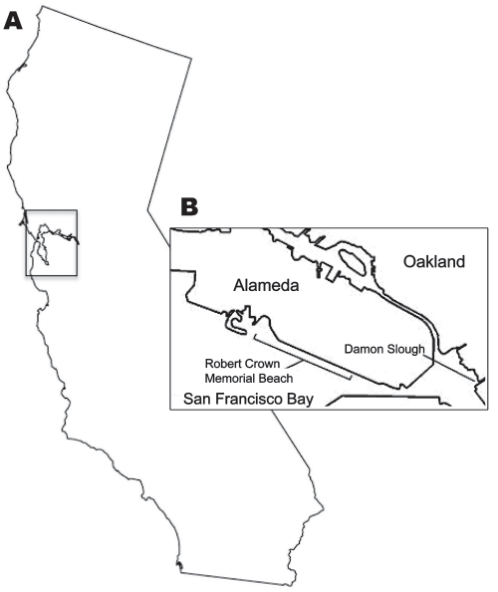
San Francisco Bay area, California, USA (A), and locations where *Haminoea japonica* snails were obtained (B).

**Figure 2:**
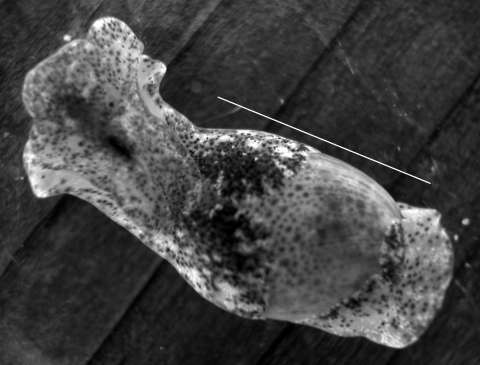
*Haminoea japonica* snail*.* Scale bar = 11 µm.

This study was conducted under the University of New Mexico Institutional Animal Care and Use Committee Protocol 07UNM011, Animal Welfare Assurance # A4023-01. Samples of *H. japonica* snails were collected by hand at low tide from 4 locations (Figure 1): Crab Cove at the northern end of Robert Crown Memorial Beach (37°46′4.18′′N, 122°16′39.55′′W) in 2005–2008; the southern end of this beach (37°45′8.81′′N, 122°14′57.33′′W) in 2006 and 2008; Damon Slough (37°45′14.12′′N, 122°12′21.6′′W) in 2007; and Lake Merritt, a tidal lagoon connected to San Francisco Bay (37°48′22.25′′N, 122°15′22.81′′W) in 2008. All gastropod species observed at Robert Crown Memorial Beach were collected and examined for cercariae. *H. japonica* (Figure 2), *Philine* sp. (Cephalaspidea: Philinidae), and *I. obsoleta* snails (all exotic) were collected on tide flat sediments. The exotic oyster drill *Urosalpinx cinerea* (Say 1822) (Sorbeoconcha: Muricidae) and the native periwinkle *Littorina* spp. (*L. scutulata* Gould 1849 or *L. plena* Gould 1849) were collected from exposed bulwarks and other hard substrates. In addition, we examined native *H. virescens* Sowerby 1833 snails collected in August 2005 from Friday Harbor, San Juan Island, Washington.

Snails were isolated singly or in groups of 5 in plastic containers in saline water (20–35 parts per thousand [ppt]) and placed in natural light to induce cercarial shedding. For each species, a subset of the larger snails was then dissected. Cercariae were photographed, and ethanol-preserved specimens were measured and compared with published descriptions of schistosome cercariae.

Gulls are common hosts for schistosomes transmitted by marine snails. We examined 29 gulls of 4 species collected at the Oakland International Airport, ≈7.5 miles southeast of Robert Crown Memorial Beach, as part of the airport’s Wildlife Management Program. For each bird, the mesenteric veins were examined, the intestine was opened, the muscosa was removed, and a sample from the intestinal wall was placed between 2 glass slides and examined for schistosome adults or eggs in the villi (*23*). Feces were screened for eggs.

### Sequencing and Phylogenetic Analysis

DNA was extracted from fresh or ethanol-preserved cercariae, amplified by PCR (Takara Ex Taq; Takara Biomedicals, Otsu, Japan), and sequenced by using published primers (*24**,**25*). PCR products were purified on Montage Microcon columns (Millipore, Billerica, MA, USA). Sequencing reactions were performed by using the BigDye Direct Sequencing Kit Version 3.1 (Applied Biosystems, Foster City, CA, USA).

Phylogenetic analyses of schistosomes obtained from *H. japonica* snails were performed for 2 datasets. The first dataset combined 18S and 28S rRNA sequence data to place our samples of *H. japonica* within the larger context of the family *Schistosomatidae* (*24*). The second dataset, which was composed of part of the internal transcribed spacer region 2, focused on taxa within the schistosome BTGD clade, which includes species of *Bilharziella*, *Trichobilharzia*, *Gigantobilharzia*, and *Dendritobilharzia* (sensu 24). Phylogenetic analyses with maximum parsimony (MP), maximum likelihood (ML), and minimum evolution (ME) were conducted by using PAUP* version 4.0b10 (*26*) and Bayesian inference (BI) by using MRBAYES 3 (*27*). The jModeltest (*28*) was used to determine the most appropriate nucleotide substitution model for ML and ME analyses.

Parsimony trees were reconstructed by using heuristic searches (300 replicates). Optimal ME and ML trees were constructed from heuristic searches (300 replicates for ME, 10 replicates for ML). Nodal support was estimated by bootstrap (200 replicates) analysis and determined for the MP and ME trees by using heuristic searches. For the ML dataset, the model selected (Akaike information model) was generalized time reversible + proportion invariant + Γ. For BI of the 18S–28S rRNA dataset, the parameters were unlinked: Nst = 6 rates = gamma ngammacat = 4. For both datasets, 4 chains were run simultaneously for 5 × 10^5^ generations; the first 5,000 trees with preasymptotic likelihood scores were discarded as burning, and the retained trees were used to generate 50% majority-rule consensus trees and posterior probabilities.

### Life Cycle

**Figure 3:**
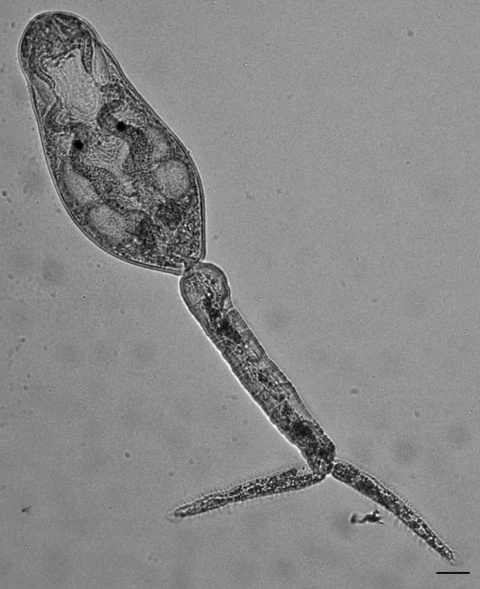
Live schistosome cercaria from a *Haminoea japonica* snail. Scale bar = 30 µm. Measurements are shown in Table 2.

To obtain adult worms for species identification, we experimentally exposed young parakeets and *Gallus gallus* L. chicks for 30 min to schistosome cercariae from *H. japonica* snails obtained from San Francisco Bay (Figure 3). Bird hosts were selected according to the method of Leigh (*23*), who described adult worms derived from cercariae from *H. antillarum guadalupensis* Sowerby snails in Florida. In experiment 1, eight chicks were exposed by standing each chick in salt water (35 ppt) containing cercariae. In experiment 2, six parakeets were exposed by applying cercariae to their bare abdomens. In experiment 3, eight parakeets were exposed by standing each bird in salt water (35 ppt) containing cercariae.

## Results

### Specimens Collected

We identified 1 species of schistosome in *H. japonica* snails that had a prevalence of 1.2% as determined by observation of shedding and 8.7% as determined by dissection (Table 1). No schistosomes were found in other snails examined, including *Littorina* spp. and *I. obsoleta*, taxa that are known to host schistosomes in California (*1**,**13*). No other trematodes were found in *H. japonica* snails from San Francisco Bay or *H. virescens* snails from Washington State. *A. variglandis* was the only schistosome found in the gulls; 55% had adult worms in their mesenteric veins (Table 1).

**Table 1:** Hosts examined for avian schistosomes at 2 locations, United States*

Animal, location, and date	Species	No. screened	No. positive	No. dissected	No. positive
Snails					
San Francisco Bay, California					
2005 Jun	*Ilynassa obsoleta*	96	0	48	0
2005 Jul	*Haminoea japonica*	672	0	300	8
	*I. obsoleta*	270	0	50	0
	*Urosalpinx cinerea*	220	0	0	0
	*Littorina* sp.	275	0	50	0
2006 May	*H. japonica*	400	0	100	0
2006 Jun	*H. japonica*	300	0	150	0
2006 Nov	*H. japonica*	222	0	100	0
2007 Jun	*H. japonica*	930	0	350	11
2007 Jul	*H. japonica*	655	0	266	37
	*Philine* sp.			38	0
2008 Aug	*H. japonica*	1,100	50	180	70
	*I. * *obsoleta*	400	0	100	0
	*Littorina* sp.	200	0	100	0
	*Philine* sp.	100	0		
San Juan Island, Washington					
2005 Aug	*H. virescens*	717	0	215	0
Birds					
San Francisco Bay, California					
2007 Aug	*Larus californicus*	NA	NA	4	2
	*L. occidentalis*	NA	NA	10	4
	*L. delawarensis*	NA	NA	1	1
2008 Jan	*L. occidentalis*	NA	NA	5	3
	*L. glaucescens*	NA	NA	1	0
2008 Mar	*L. occidentalis*	NA	NA	7	5
	*L. glaucescens*	NA	NA	1	1

*NA, not applicable.

Cercariae, most of which were collected by dissection, lay in contact with the surface film of water, where they were mostly inactive except for occasional tail twitching. The cercariae were apharyngeate with pigmented eyespots, a lightly spined body, dorsal and ventral fin folds on the full length of the tail furcae, 5 pairs of flame cells, and 3 pairs of penetration glands (Figure 3). With respect to these behavioral and morphologic features, and on the basis of size, the cercariae most closely resemble those of *Gigantobilharzia huttoni* (Leigh 1953), the only other schistosome previously collected from haminoeid snails (*23*,*29*) (Table 2). These cercariae differ from those of *G. huttoni* in having fewer pairs of penetration glands (3 pairs instead of 5–6 pairs), but this trait is difficult to discern accurately. In contrast, *A. variglandis* cercariae have 6 pairs of penetration glands and 6 pairs of flame cells, are larger, and have different proportions than cercariae we found (Table 2). Specimens of the schistosome obtained from *H. japonica* snails were deposited in the Parasite Division of the Museum of Southwestern Biology at the University of New Mexico (MSB185).

**Table 2:** Characteristics of selected schistosome cercariae from 5 locations, United States*

Characteristic	Schistosome					
Species	Unidentified	*Gigantobilharzia huttoni*	*G*. *huronensis*	*Austrobilharzia variglandis*	*A. variglandis*	*A. variglandis*
Number	14	30	50	?	10	232
Fixative	Alcohol	Formalin	Formalin	Bouin solution or hot corrosive sublimate fluid	Formalin	Formalin
Host species	*Haminoea japonica*	*H*. *antillarum guadalupensis*	*Physa gyrina*	*Ilyanassa obsoleta*	*I. obsoleta*	*I. obsoleta*
Collection location	San Francisco Bay, CA	Virginia Key, Miami, FL	Ann Arbor, MI	Quamquissett Harbor, MA	Rhode Island	San Francisco Bay, CA
Body length	188 ± 4.8 (160–216)	152 ± 1.4 (135–164)	240 (195–270)	262	237 (220–250)	258 (241–275)
Body width	58 ± 1.3 (50–64)	35 ± 0.7 (48–64)	72.5 (63.7–105)	77	72.5 (69.0–79.0)	–
Tail stem length	175 ± 3.3 (155–197)	154 ± 1.2 (140–166)	268.5 (255–300)	157	228 (200–236)	286 (264–315)
Tail stem width	24 ± 0.7 (21–29)	19 ± 0.3 (16–22)	30 (22.5–45)	–	22–36	–
Furca length	78 ± 2.6 (60–92)	79 ± 0.8 (72–86)	146.8 (102.5–172.5)	105	126 (120–140)	190 (161–216)
Furca width	13 ± 0.6 (10–16)	10 ± 0.4 (6–13)	15	–	12.0–16.5	–
Oral sucker length	57 ± 1.9 (49–70)	63 ± 0.6 (54–70)	–	–	72 (66–85)	–
Oral sucker width	46 ± 0.7 (43–51)	53 ± 0.7 (48–64)	–	–	43 (40–45)	–
Ventral sucker to posterior end	54 ± 3.3 (35–76)	44 ± 0.1 (35–51)	–	–	–	–
Body length:tail stem length	1.1	0.99	0.89	1.7	1	0.9
Tail stem length:furca length	2.2	1.9	1.8	1.5	1.8	1.5
Pairs of flame cells†	4 + 1	4 + 1	4 + 1	5 + 1	5 + 1	–
Pairs of penetration glands	3	5–6	5	6	6	–
Reference	This report	(*24*,*29*)	(*30*)	(*31*)	(*14*)	(*1*)

*Values are mean ± SE in microns (range), or range only, unless otherwise indicated. For Miller and Northup (*31*), the tail stem and furca lengths were estimated values based on information in the text. For Grodhaus and Keh (*1*), the ranges were mean values for 7 lots of cercariae obtained from 7 snails. †No. pairs in body plus no. pairs in tail.

### Sequencing and Phylogenetic Analysis

**Figure 4:**
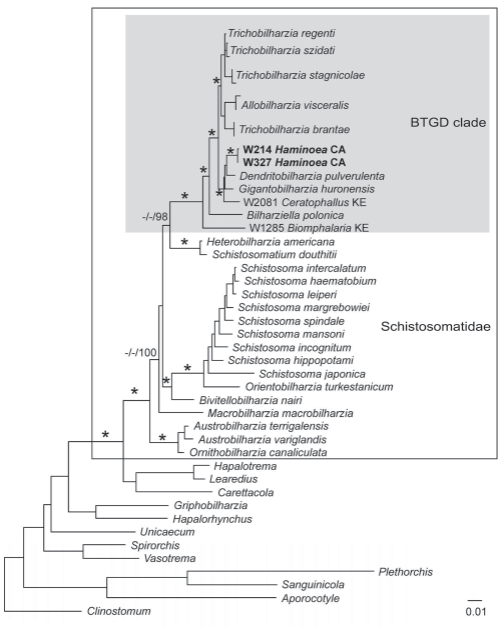
Maximum-likelihood phylogenetic tree based on 18S–28S rRNA sequences of schistosomes. Schistosomatids are indicated in the large box and the *Bilharziella, Trichobilharzia, Gigantobilharzia,* and *Dendritobilharzia* (BTGD) clade is indicated in the gray box. Samples in **boldface** are those obtained from *Haminoea japonica* snails. Node support is indicated by maximum parsimony (MP) and minimum evolution (ME) bootstrap values and Bayesian posterior probabilities (PPs), respectively. Asterisks indicate MP and ME bootstrap values >85 and PPs >98 and hyphens indicate no significant node support. Branch support is designated only for major clades. Scale bar indicates nucleotide substitutions per site.

**Figure 5:**
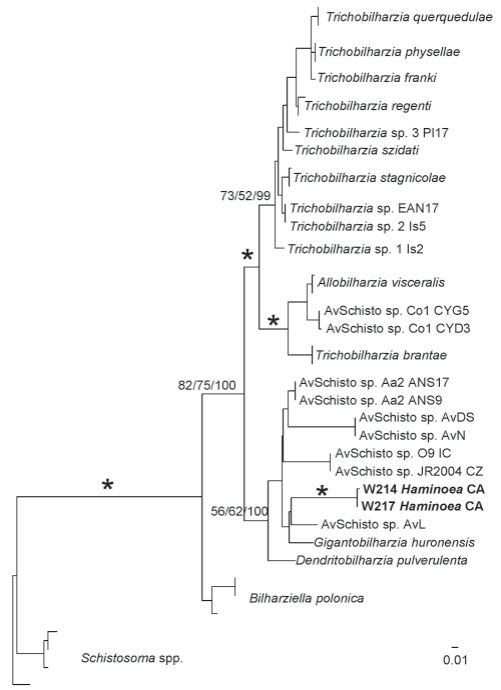
Maximum-likelihood phylogenetic tree based on internal transcribed spacer region 2 sequences of relationships among members of the *Bilharziella, Trichobilharzia, Gigantobilharzia,* and *Dendritobilharzia* species clade from this study and unidentified samples of avian schistosomes from GenBank (online Appendix Table, www.cdc.gov/EID/content/16/9/1357-appT.htm). Samples in **boldface** are those obtained from *Haminoea japonica* snails. Node support is indicated by maximum parsimony (MP) and minimum evolution (ME) bootstrap values and Bayesian posterior probabilities (PPs), respectively. Asterisks indicate MP and ME bootstrap values >85 and PPs >98. Branch support is designated only for major clades. Scale bar indicates nucleotide substitutions per site.

Schistosome taxa used in the phylogenetic analyses are shown in the Table A1. Cercariae from *H. japonica* were distinct from all other available schistosomes (GenBank accession nos. GQ920617–21). These cercariae belong to the BTGD clade, which contains only freshwater schistosomes (Figure 4). Our internal transcribed spacer region 2 dataset includes all reported schistosomes from GenBank that belong to the BTGD clade (Figure 5). Only an ML tree is shown in Figure 5. However, MP, ME, and BI analyses yielded near identical topologies with differences at the tips and at nodes where there is no clade support.

### Life Cycle

Three experiments with birds were conducted to obtain adult worms. However, all birds experimentally exposed were negative for schistosome infection.

## Discussion

Cercarial morphology and molecular genetic data for the schistosome from *H. japonica* indicate that these cercariae are not *A. variglandis*, a schistosome previously reported in San Francisco Bay, and the only species previously implicated in dermatitis outbreaks on the Pacific Coast. We obtained *A. variglandis* schistosomes from gulls but not from snails in the San Francisco Bay area. The *H. japonica*–transmitted schistosome is the second species reported to cause dermatitis after introduction of an exotic snail in California coastal waters. We found this schistosome in an opisthobranch snail in western North America. The only other schistosome known to be obtained from an opisthobranch snail is *G. huttoni* from *H. a. guadalupensis* snails obtained in Florida (*23**,**29**,**32*). Except for schistosomes collected from 2 species of *Siphonaria* (Pulmonata: Siphonariidae) snails (*33**,**34*), all other marine schistosomes have been obtained from caenogastropodid snails.

Cercariae from *H. japonica* closely resemble those of *G. huttoni*, for which sequence data are not available. DNA sequence data for cercariae from *H. japonica* did not match with those of any known schistosome species, including the congener *G. huronensis* Najim 1950. Cercariae from *H. japonica* did not group with other marine schistosomes, but belong to the BTGD clade that, until now, included only freshwater species that use pulmonate snails as intermediate hosts (Figure 5). The only other known marine schistosomes belong to species of the genera *Austrobilharzia* and *Ornithobilharzia* Ohdner 1912, which are distantly related to the BTGD clade (Figure 4). We exposed parakeets and chicks to cercariae from *H. japonica* snails but were unable to obtain adult worms for comparison with described species.

Schistosomes from *H. japonica* and those of *G. huttoni* are probably closely related because they are found in haminoeid snails and have morphologically similar cercariae. However, they differ from all other species in the genus *Gigantobilharzia* Ohdner 1910 in habitat (salt water rather than fresh water) and snail host (opisthobranchid rather than pulmonate). When appropriate genetic material becomes available, analyses may show that *G. huttoni* schistosomes and those from *H. japonica* snails should be placed in a separate genus.

*Gigantobilharzia* spp. have been reported in several gull species (*35*). Gulls are common at Robert Crown Memorial Beach, often resting on the beach flats at low tides and sometimes foraging in tide pools that contain *H. japonica* snails (A.N. Cohen, unpub. data). Gulls are thus a likely host for schistosomes from *H. japonica*, although we did not find them in gulls at the Oakland Airport near Robert Crown Memorial Beach. Leigh (*36*) and Kinsella et al. (*37*) found fragments of adult worms that they identified as *Gigantobilharzia* sp., and which closely resembled *G. huttoni* worms, in pelicans in Florida. Small flocks of brown pelicans and, rarely, white pelicans have been observed in shallow water off Robert Crown Memorial Beach, although not on the beach or in tide pools (A.N. Cohen, unpub. data). Pelicans are thus another possible host for schistosomes from *H. japonica*.

Other birds commonly observed at Robert Crown Memorial Beach include shorebirds that are most common in winter when water temperatures are probably less conducive to cercarial emergence (surface temperatures near this beach are typically 16°C–20°C in summer and 8°C–12°C in winter). Cormorants, grebes, and ducks are found in near-shore waters, and mallard ducks sometimes forage in tide pools. Larger wading birds (snowy and great egrets, and occasionally herons), oystercatchers, and several species of terns are sometimes seen in small numbers foraging on the beach or in shallows. Marine schistosomes have been reported in gulls, ducks, terns, herons, cormorants, and turnstones (*14**,**17**,**19**,**23*), and *Gigantobilharzia* spp. have been reported in grebes and cattle egrets (*23**,**35*). Thus, various bird species might serve as hosts for schistosomes from *H. japonica*.

Cercarial dermatitis is commonly acquired in fresh water (*38*). It is less common in marine or estuarine waters; most cases are reported from the northwestern Atlantic Ocean or Australia. This disease was observed on the Pacific Coast of North America during an outbreak at Robert Crown Memorial Beach in 1954–1956, when cercariae identified as *A. variglandis* were found in *I. obsoleta*, an Atlantic snail introduced before 1907. The schistosome was likely introduced with this snail and remained undetected until the 1950s (*1*). In June 2005, cercarial dermatitis was again reported at Robert Crown Memorial Beach. Initial cases were found among elementary school groups that visited the beach at the end of the academic year. Since 2005, dermatitis has occurred annually at this beach (90 cases in 2005, 3 in 2006, 14 in 2007, and 31 in 2008).

Schistosome cercariae in *H. japonica* snails, large numbers of these snails at Robert Crown Memorial Beach, and the apparent absence of schistosomes in other common snails at this site indicate that schistosomes from *H. japonica* are responsible for the recent dermatitis outbreak. *H. japonica* snails were first seen in California in 1999 (*22*) and except for the 1954 outbreak attributed to *A. variglandis*, cercarial dermatitis was not reported in San Francisco Bay until shortly after the arrival of *H. japonica*.

**Figure 6:**
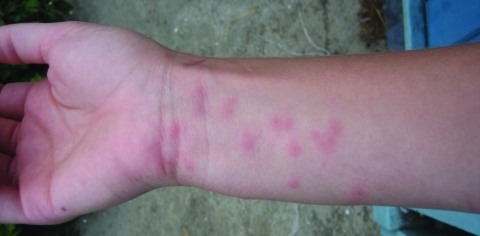
Cercarial dermatitis contracted in San Francisco Bay, California, USA, by one of the authors (S.V.B.).

The most popular water-contact activities at Robert Crown Memorial Beach are kite surfing and wind surfing at high tide and wading, exploring, and playing in shallow pools at low tide. In part because of cool water temperatures at this beach, swimming is uncommon, and kite and wind surfers usually wear wetsuits. Most dermatitis cases at this beach were contracted by persons wading in tide pools. Dermatitis usually occurred on the feet or legs. Only 1 case was reported among kite surfers and wind surfers. Since 2005, three biologists working at this beach have contracted dermatitis, usually on their hands or forearms (Figure 6). In experiments with *G. huttoni* schistosomes obtained near Miami, Florida, cercariae emerged only when temperatures were >22°C, regardless of season or light intensity (*32*). In San Francisco Bay, the highest numbers of cercariae from *H. japonica* may be released in beach flat tide pools that warm up during daytime low tides.

There are at least 3 ways in which schistosomes we found in *H. japonica* snails could have recently emerged as a disease agent in San Francisco Bay. First, an unknown native schistosome may be present in native birds and snails, which switched hosts to the introduced *H. japonica* snail. Some evidence shows that schistosomes are capable of switching hosts (*39*). Neither of 2 native *Haminoea* species found in western North America between Baja, California and Alaska (*H. vesicula* [Gould 1855] and *H. virescens*) (*22*) is known to host schistosomes or is found in the study area. However, if an undetected native schistosome is present in 1 of these species, or in a *Haminoea* species further south on the Pacific Coast, migration of its bird host over San Francisco Bay could have resulted in infection of the *H. japonica* population at Robert Crown Memorial Beach, greatly increasing the potential for human dermatitis. The range of *G. huttoni* schistosomes may extend from Florida through the Caribbean and (by bird movements) up the Pacific Coast.

Second, the schistosomes could be a species from Asia recently introduced into San Francisco Bay in infected *H. japonica* snails. This introduction would require adult snails (eggs and larvae do not contain schistosomes) that harbor male and female cercariae; both sexes would have to colonize the same bird to initiate egg-producing infections. This event occurred on 1 occasion when *A. variglandis* schistosomes and *I. obsoleta* snails were introduced into western North America. Whether *H. japonica* snails arrived in San Francisco Bay directly from Asia or indirectly through Washington State is unknown. *H. japonica* snails may have been introduced into Washington State, where they were first observed in the early 1980s (*40*), in Pacific oysters (*Crassostrea gigas* Thunberg 1793) imported from Japan for mariculture (*17*). Pacific oysters from hatcheries or oyster farms in Japan, Washington, Oregon, and California were placed in San Francisco Bay for commercial mariculture in the 1930s, for occasional experimental use until 1981, for bioaccumulation studies during 1991–2002, and were introduced illegally at 1 site in 1999. A population recently discovered in South San Francisco Bay appears to have been introduced during the late 1990s (A.N. Cohen, D. Goodwin, unpub. data). These occurrences may be related to the initial appearance of *H. japonica* snails in 1999 in South San Francisco Bay.

Third, the schistosomes could be a species from Asia found in migrating birds that infected *H. japonica* snails after these snails became established. Because some birds excrete schistosome eggs for <10–28 months postinfection (*19**,**23*), some schistosomes may survive in a bird host long enough to complete a long-distance migration. However, because no bird species are known to routinely migrate across the Pacific Ocean between the native region of *H. japonica* snails in Asia and regions in the western United States, introduction by this mechanism seems unlikely.

Much remains to be learned about factors favoring outbreaks of cercarial dermatitis in new areas. Native *Haminoea* spp. should be surveyed for parasites to assess whether host switching may be involved, and *H. japonica* snails should be surveyed in their native range and in Washington State to determine whether trans-Pacific schistosome colonization events have occurred and by what mechanisms. Molecular analysis of *G. huttoni* schistosomes would increase the taxonomic status of the species we isolated from San Francisco Bay. The definitive avian host in this region could be determined by examination of feces for eggs and carcasses for adult schistosomes.

The molecular signatures we have provided may be present in schistosomes isolated from birds or snails in other areas, which would help establish how this zoonotic infection reached California. Potential effects on native biota, especially endangered birds that might serve as hosts (such as the California least tern or California brown pelican), should be assessed. Whether this schistosome will become established in other locations along the Pacific Coast and affect beach users is unknown. Improved understanding of the biology and mechanism of establishment of this schistosome may enable better management of human exposure and infection, control of its spread, and prevention of other schistosome introductions or outbreaks.

## Appendix

**Table A1:** Taxa used in the phylogenetic analysis of schistosomes, United States*

Taxa	Host	Gene region		
		18S rRNA	28S rRNA	ITS
New marine schistosome				
W214 *Haminoea* CA	*Haminoea japonica*	GQ920617	GQ920619	GQ920622
W217 *Haminoea* CA	*H. japonica*			GQ920621
W327 *Haminoea* CA	*H. japonica*	GQ920618	GQ920620	
*Allobilharzia visceralis*				DQ067561
W246 tusw NV	*Cygnus columbianus*	EF114220	EF114222	EF071990
*Austrobilharzia terrigalensis*		AY157223	AY157249	
*Austrobilharzia variglandis*		AY157224	AY157250	
*Bilharziella polonica*	*Anas platyrhynchus*	AY157214	AY157240	EF094539, FJ793899, FJ793898
*Dendritobilharzia pulverulenta*	*Gallus, Mergus*	AY157215	AY157241	EF071988
*Gigantobilharzia huronensis*	*Agelaius phoeniceus*	AY157216	AY157242	EF071987
*Macrobilharzia macrobilharzia*		AY829260	AY858885	
*Ornithobilharzia canaliculata*		AY157222	AY157248	
*Trichobilharzia brantae*				
W340 sngo MB	*Chen caerulescens*	FJ174451	FJ174467	FJ174533
W330 *Gyraulus* CO	*Gyraulus parvus*	FJ174450	FJ174466	FJ174532
*Trichobilharzia franki*	*Radix auricularia*	FJ711767	FJ711768	AY713969
*Trichobilharzia physellae*				
W146 *Physa* NM	*Physa gyrina*			FJ174568
W255 buhe NM	*Bucephala albeola*	FJ174458	FJ174474	FJ174561
*Trichobilharzia querquedulae*				
W190 blte CA	*Anas discors*			FJ174550
W180 cite CA	*Anas cyanoptera*	FJ174454	FJ174470	FJ174556
*Trichobilharzia regenti*	*Radix peregra*	AY157219	AY157245	EF094533
* Trichobilharzia* sp. EAN35	*R. peregra*			EU413974
*Trichobilharzia stagnicolae*				
W240 come MI	*Mergus merganser*	FJ174462	FJ174478	FJ174544
W164 *Stagnicola* NM	*Stagnicola* sp.	FJ174461	FJ174477	FJ174540
*Trichobilharzia szidati*				
Blind sucker *Lymnaea* MI	*Lymnea stagnalis*	FJ174460	FJ174476	
Flathead *Stagnicola* MT	*Stagnicola elrodi*			FJ174539
* T. szidati*	*L. stagnalis*	AY157219	AY157245	EF094541
Unspecified species of *Trichobilharzia*				
* Trichobilharzia* sp. 1 Is2	*R. peregra*			FJ469784
* Trichobilharzia* sp. 2 Is5	*R. peregra*			FJ469792
* Trichobilharzia* sp. 3 Pl17	*Anas penelope*			EF094532
* Trichobilharzia* sp. EAN17	*R. peregra*			EU413971
Unidentified species of avian schistosomes				
AvSchisto sp. AvDS	*Anisus vortex*			FJ786029
AvSchisto sp. AvL	*A. vortex*			FJ786030
AvSchisto sp. AvN	*A. vortex*			FJ786028
AvSchisto sp. 09 IC	*R. peregra*			FJ469822
AvSchisto sp. JR2004 CZ	*Physa fontinalis*			AY713963
AvSchisto sp. Aa2 ANS17	*Anser anser*			FJ793909
AvSchisto sp. Aa2 ANS9	*A. anser*			FJ793908
AvSchisto sp. Aa3 ANS36	*A. anser*			FJ793914
AvSchisto sp. Co1 CYG5	*Cygnus olor*			FJ793921
AvSchisto sp. Co1 CYD3	*C. olor*			FJ793920
W2081 *Ceratophallus* KE	*Ceratophallus* sp.	AY829259	AY858887	
W1285 *Biomphalaria* KE	*Biomphalaria sudanica*	AY829258	AY858886	
Mammalian schistosomes				
* Schistosomatium douthitti*		AY157221	AY157247	
* Heterobilharzia americana*		AY157220	AY157246	
* Schistosoma intercalatum*		AY157235	AY157262	
* S. hematobium*		Z11976	AY157263	
* S. leiperi*		AY157234	AY157261	
* S. margrebowiei*		AY157233	AY157260	
* S. spindale*		Z11979	AY157257	
* S. incognitum*		AY157229	AY157255	
* S. hippopotami*				AY197343
* S. japonicum*		AY157226	AY157607	
* Orientobilharzia turkestanicum*		AF442499	AY157254	
* Bivitellobilharzia nairi*		AY829261	AY858888	
Outgroup taxa				
* Hapalotrema mehrai*		AY604716	AY604708	
* Learedius learedi*		AY604715	AY604707	
* Carettacola hawaiiensis*		AY604717	AY604709	
* Hapalorhynchus gracilis*		AY604718	AY604710	
* Griphobilharzia amoena*		AY899915	AY899914	
* Unicecum* sp.		AY604719	AY604711	
* Vasotrema robustum*		AY829257	AY858883	
* Spirorchis scripta*		AY829256	AY858882	
* Plethorchis acanthus*		AY222096	AY222178	
* Aporocotyle spinosicanalis*		AJ287477	AY222177	
* Clinostomum* sp. USA		AY222095	AY222095	

*ITS, internal transcribed spacer. GenBank accession numbers are indicated.
